# Compliance of WHO Surgical Safety Checklist at a Pediatric Surgical Unit in a Tertiary Level Hospital: A Descriptive Cross-sectional Study

**DOI:** 10.31729/jnma.7045

**Published:** 2021-12-31

**Authors:** Jasmine Bajracharya, Ritesh Shrestha, Deepika Karki, Asim Shrestha

**Affiliations:** 1 Pediatric Surgery Unit, Department of Surgery, Nepal Medical College and Teaching Hospital, Jorpati, Kathmandu, Nepal; 2 Nepal Medical College and Teaching Hospital, Jorpati, Nepal

**Keywords:** *checklist*, *compliance*, *critical medical incidents*, *never events*, *surgical errors*

## Abstract

**Introduction:**

The surgical safety checklist by World Health Organization has been used for the last two decades. There is every chance of unwanted expected disasters in operating-room in pediatric-surgical cases. Our study is to observe the utilization of the safety checklist in a tertiary level pediatric surgery unit in Nepal.

**Methods:**

A descriptive cross-sectional study was done at Nepal Medical College Teaching Hospital from January 2021-June 2021 with record-based data of children from 0-15 years operated in Pediatric Surgery unit from March 2017-July 2018. Ethical approval (Reference number: 049-077-078) was taken from the Institution review committee of the institute. Convenience sampling was done. Selfdesigned Pro-forma with demographic data along with World Health Organization-Surgical-safety-checklist used was collected and entered in Microsoft-Excel. Data were analyzed using Statistical-Package-for-the-Social-Sciences-version-25. Point estimate at 95% Confidence Interval was done along with frequency and proportion for binary data.

**Results:**

Out of 267 cases enrolled, 103 (38.6%) (35.6-41.6 at 95% Confidence Interval) were fully compliant with the checklist, 69 (25.8%) partially compliant. Among compliant cases, 148 (55.4%) Sign-in part, 128 (47.9%) cases -Time-out part and 152 (56.9%) cases Sign-out part were complete.

**Conclusions:**

Compliance with World Health Organization-Surgical-safety-checklist has a major role in preventing morbidity and mortality in Pediatric surgical cases. With proper use of the checklist, the unwanted never-events can be prevented with better surgical outcomes.

## INTRODUCTION

World Health Organization (WHO) had launched the Safe Surgery Saves Life campaign in 2007, to improve consistency in surgical care and adherence to safety practices.^1^ They had launched surgical safety checklist in 2008 first and revised it in 2009. There has been increased awareness of need to improve quality of health-care, over past two decades, regardless of income or level of development.^2^

Surgical services in institutes with multidisciplinary faculties have variety of cases with different age groups in single pre-operative area. So there is every chance of mistaken identity of the patient, diagnosis and procedure. Wrong pre-medication, entry in operation-theatre and handling by the team is one of the unwanted expected disasters.

This study is to observe utilization of the safety checklist with its full, partial or non-compliance in pediatric surgery cases in a tertiary level pediatric surgery unit in Nepal.

## METHODS

This is a descriptive cross-sectional study carried out in Nepal Medical College and Teaching Hospital, Jorpati, Kathmandu for a duration of 6 months from January 2021-June 2021 among patients aged zero days-fifteen years attending pediatric surgery unit with the help of record-based data. Ethical clearance was obtained from Institution Review Committee (IRC-NMCTH) with an archived approval number 049-077078. After explaining the objective and plan of study and fulfilling inclusion and exclusion criteria, informed written consent was taken from the guardian of the patients. All pediatric surgical patients operated on and recorded in Operation-Theater in NMCTH from March 2017-July 2018 were included in the study. Those patients operated in minor operation theater, were excluded from the study. Convenience sampling was done.

For the descriptive cross-sectional study, the minimum required sample size for the study was calculated as follows,

n = Z^2^ × p × q / e^2^

  = (1.96)^2^ × (0.5) × (1-0.5) / (0.06)^2^

  = 267

Where,

n= required sample size
Z= 1.96 at 95% Confidence Interval
p= prevalence for maximum sample size, 50%
q= 1-p
e= margin of error, 6%

Hence, 267 cases are enrolled in the study by generating random numbers in Microsoft Excel.

In the first step, a self-designed Pro-forma containing demographic data was prepared separately and utilized in all cases. The WHO Surgical Safety Checklist was obtained from the official WHO website^3^ and printed with the Pro-forma. It was used in the Pre-operative ward and Operation Theater; conducted by surgical residents and intern doctors posted in Pediatric Surgery Unit during that period. The Surgical Residents and Interns were given a brief introduction and information regarding the proper use of the checklist beforehand. The Sign-in was started in Pre-operative ward and Timeout and Sign-out were done in Operation Theater. The Checklists were collected and data of all the patients were recorded in Microsoft Excel on a regular basis.

Selection bias has been minimized as possible by generating random numbers in Microsoft Excel. The collected data was analyzed using Statistical Package for the Social Sciences (SPSS) version 25 and descriptive analysis was done. Point estimate at 95% confidence interval was calculated along with proportion and frequency for binary data.

## RESULTS

**Figure 1:**
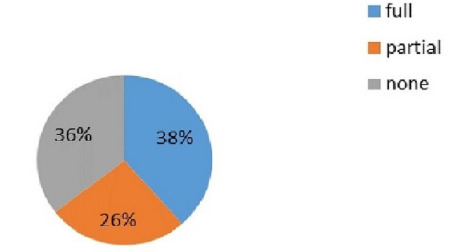
Compliance of WHO Surgical Safety Checklist.

Out of 267 cases enrolled, 103 (38.6%) (35.6-41.6 at 95% Confidence Interval) were fully compliant, 95 (35.6%) were none compliant and 69 (25.8%) cases were partially compliant (Figure 1).

**Figure 2:**
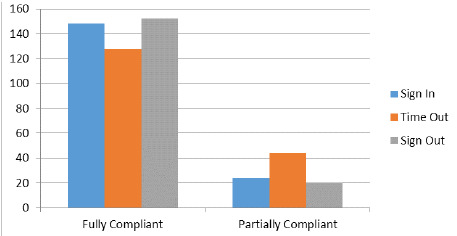
Categorization of compliance.

Among the compliant cases 148 (55.4%) had complete Sign-in part, 128 (47.9%) cases had complete Timeout part and 152 (56.9%) had complete Sign-out part. (Figure 2).

We had recorded the checklist in a paper Pro-forma where we considered important portions from each section of the checklist. In the Sign-in part confirmation of identity, site mark, known allergy and difficult airway or aspiration risk were considered. In the Time-out part, reconfirmation of identity, antibiotic prophylaxis were considered. Finally, in the Sign-out part completion of the instrument, sponge and needle counts, and equipment-related problems addressed were considered as shown in (Table 1).

**Table 1:** Completeness of Surgical Safety Checklist.

WHO Checklist	Completeness of filling of checklist	Individual items mostly missed during filling of checklist	Present n (%)	Absent n (%)	Others n (%)
Sign In	Complete 148	1. Confirmed patient identity	148 (100)	0	0
	(55.4)	2. Site marked	84 (56.8)	0	64 (43.2)
		3. Known Allergy	6 (4.1)	142 (95.9)	0
		4. Difficult airway or aspiration risk	38 (25.7)	110 (74.3)	0
	Incomplete 119	1. Confirmed patient identity	24 (20.2)	95 (79.8)	0
	(44.6)	2. Site marked	10 (8.4)	103 (86.6)	6 (5.0)
		3. Known Allergy	1 (0.8)	20 (16.8)	98 (82.4)
		4. Difficult airway or aspiration risk	4 (3.4)	17 (14.3)	98 (82.4)
Time Out	Complete	1. Confirmed patient identity	128 (100)	0	0
	128 (47.9)	2. Antibiotic Prophylaxis in last 60 minutes	122 (95.3)	0	5 (3.9) 1 (0.8)
	Incomplete	1. Confirmed patient identity	33 (23.7)	106 (76.3)	0
	139 (52.1)	2. Antibiotic Prophylaxis in last 60 minutes	37 (26.6)	0	102 (73.4)
Sign Out	Complete	1. Completion of Instrument, Sponge and needle counts	152 (100)	0	0
	152 (56.9)	2. Equipment related problems to be addressed	53 (34.9)	99 (65.1)	0
	Incomplete	1. Completion of Instrument, Sponge and needle counts	10 (8.7)	1 (0.9)	104 (90.4)
	115 (43.1)	2. Equipment related problems to be addressed	0	7 (6.1)	108 (93.9)

* NA- Not Applicable

* NR- Not Recorded

In our study, demography of the Age group and gender were as shown in the (Table 2).

**Table 2:** Demographic features.

Parameters		n (%)
**Age Group**	<28 days	6 (2.2)
	1 month-1 year	17 (6.4)
	1-5 years	101 (37.8)
	5-12 years	115 (43.1)
	>12 years	28 (10.5)
**Gender**	Male	212 (79.4)
	Female	55 (20.6)
**Age (Mean ± SD) in Years**	6.68 ± 4.26	(2.42-10.94) years

The cross tabulation of compliance and types of surgery showed full compliance observed more in elective cases 92 (49.7%) than emergency cases 11 (13.4%) and none compliance more in emergency 53 (64.6%) than elective cases 42 (22.7%). But there was not a noticeable pattern in major, intermediate or minor cases as seen in (Table 3).

**Table 3:** Cross-tabulation of Compliance and Types of Surgery.

Type of Surgery	Full Compliance n (%)	Partial Compliance n (%)	None n (%)
Elective	92 (49.7)	51 (27.6)	42 (22.7)
Emergency	11 (13.4)	18 (22)	53 (64.6)
Major	23 (29.5)	20 (25.6)	35 (44.9)
Intermediate	55 (45.5)	33 (27.3)	33 (27.3)
Minor	25 (36.8)	16 (23.5)	27 (39.7)

There were no postoperative complications in cases with full compliance but there were post-operative complications in cases with partial 9 (3.4%) and none 9 (3.4%) compliance as seen in (Table 4).

**Table 4:** Cross-tabulation of Compliance and Post-op complication.

	Presence of Post-operative Complication n (%)	Absence of Post-operative Complication n (%)
Full Compliance	0 (0)	103 (38.6)
Partial Compliance	9 (3.4)	60 (22.5)
None	9 (3.4)	86 (32.2)
Total	18 (100)	249 (100)

We did not encounter any cases with never events (wrong patient, wrong site, or wrong procedure) during the period of the study.

## DISCUSSION

WHO surgical safety checklist was implemented in our institute in Pediatric surgery cases, which showed compliance of the team members with the checklist and its outcome. Among 267 cases enrolled in the studies, full compliance was seen in 103 (38.6%) of the cases. Among these, Sign-in was complete in 148 (55.4%) cases, Time-out was complete in 128 (47.9%) cases and Sign-out was complete in 152 (56.9%) cases.

In a study by Vogts, et al. it was found that, the rate of checklist domain administration for Sign In - 99, Time Out-94 and Sign Out-2 per100 cases. The mean(range) checklist item compliance was found as 56% (27-100%) for Sign In, 69% (33-100%) for Time out, and 40% for Sign Out. Patient identity and surgical procedure were administered in 100% of Sign In and the timing of checklist administration was appropriate in 80% of cases.^4^ In another study by Yu, et al. completion rates at the four study sites were acceptable in the Sign-in stage (80.4-100%), passable in the Time-out stage (40.0-88.8 %) and poor in the Sign-out stage (10.2-59.5%).^5^ In another study, Sign-in and Time-out period were performed in a satisfactory manner yet it was not performed with equal frequency in all aspects of the items.^6^

Among the cases where a checklist was used, in the Sign-in portion, the Patient's identity was confirmed in all the cases with complete checklists. It was missing in 95 cases where the checklist was incomplete. The patient identification was done in the pre-operative area by confirming the patient's name with the parents or the informant attending the child as well as the Patient identification tags in the child's wrist. The site was marked in 84 (56.8%) cases where it was applicable. It was not applicable in 64 (43.2%) cases and it was not marked in 103 cases where it was applicable. There were no known allergies in 142 (95.9%) cases and only 6 (4.1%) cases had known allergies that were considered during the procedure. It was not recorded in 98 cases. Time out portion was completed in 128 (47.9%) cases and incomplete in 139 (52.1%) cases. Among the completed cases, all the patients' identity was reconfirmed and it was missing in 106 cases. Antibiotic prophylaxis was given in 159 cases whereas it was not recorded in 103 cases. In Sign-out, 152 (56.9%) was complete and 115 (43.1%) was incomplete. Instruments, sponge, and needle counts were completed in 162 cases and not recorded in 104 cases. Equipment-related problems were recorded in 53 cases, which was absent in 99 cases and not recorded in 108 cases.

In our study sign-out was mostly omitted, as seen in the study of Vogts, et al. where they also found that the Sign Out domain was almost always omitted, which increased the risk of omissions in postoperative care.^4^ Similar finding was seen in another study hence, the Sign-out section was clearly seen as more difficult, and less important, to complete than other sections.^6^ A study found patients having children with the same name and identical surgical procedures posted in the same operation list with patient's identification tags missing and the side of the procedure not mentioned. Mentioning of the side of operation was mixed up in case papers and consent forms. Antibiotic orders were not mentioned. Immobilization of the patients was suboptimal, leading to the displacement of the diathermy ground pad. The checklist was not used in 54 (1.8%) of cases and incompletely filled in 76 (2.5%). This shows adherence to the checklist helps to detect the instance of human error and equipment malfunction. It identifies areas needing strengthening and streamlining as in our study.^7^

Full compliance was seen more in elective cases than in emergency cases, in our study. The checklist non-compliance was seen more in emergency cases (64.6%). More intermediate cases were done and full compliance was seen in those cases. So, the type of surgery might act as a confounding variable in the study for compliance with the checklist. In a study done in a teaching hospital in Nepal, modified SSC was used in 50.5% routine and 49.5% emergency cases. Among which 80% compliance was found in routine cases and 55% in emergency cases. Poor compliance due to ignorance of the use of SSC, emergency nature of the procedure, and change of staff was noticed. Seventy percent of cases had full completion of checklist with Sign out part mostly left, similar to our study.^8^

We did not find any noticeable post-operative complications in cases with full compliance. There were a total of 18 cases with postoperative complications, which were seen in cases with partial (3.4%) and none (3.4%) compliance. Most of the post-operative complications were SSI during the postoperative period and internal fluid collections which were managed during the hospital stay of the patients. Abbott, et al. found that the incidence of postoperative complications and death in patients exposed to a surgical safety checklist was lower compared to patients who were not exposed to the checklist but it remains uncertain whether these associations were a direct causal effect, or if it simply reflected the wider quality of care in hospitals where the use of checklist was routine, as seen in our study.^9^

During the period of the study, we did not encounter any cases of a wrong patient, wrong site, and wrong procedure. This may have been due to the use of the checklist during that period. Even in the cases with non-compliance, the patient's identity, sites, and procedure were carefully checked during the study period. This might have been a Hawthorne effect due to consciousness of the use of the checklist. Compliance with the checklist offers opportunities to the surgical team, to make the workplace a more respectful and happier environment with professionalism and appropriate behavior of champions.^10^

A review by Cadman, et al. revealed that the literature in low and middle-income countries, in which results identify a lack of available literature specific to developing countries, the greatest impact could potentially be observed where lack of research was attributed to lack of resources, infrastructure, and funding to undertake the necessary research.^11^

It has been demonstrated that an educational implementation strategy can be used based on prior pilot studies utilizing lectures, film, small group breakouts, participant feedback, and simulation to teach the knowledge, skills, and behavior. Use of this course for checklist implementation resulted in 78% of participants using the checklist, at three months.^12^ In our study, the surgical residents and the interns along with the nursing staff were given a brief introduction of the checklist, on how to coordinate and use the checklist prior to the procedure. This helped in the smooth conduction of the implementation of the checklist in the study period with a favorable outcome.

There are few limitations in our study, as a small sample size from a single institute. Here, partial compliance was seen in 69 (35.6%) cases, where the checklist used, was incomplete. Sign-in was incomplete in 24 cases, time-out was incomplete in 44 cases and sign-out was incomplete in 20 cases. This may be due to lack of co-operation by the team members with the person conducting the checklist or maybe due to lack of time and coordination during the procedure. Noncompliance was seen in 95 (35.6%) of cases. This may be due to ignorance of the team members or may have been missed during emergency cases due to lack of time or manpower during those cases. The cases were taken from recorded data, so patients were followed only when they were under our supervision. This data cannot be generalized in the general population as only the pediatric patients attending our hospital were considered in our study.

## CONCLUSIONS

WHO surgical safety checklist has a major role in minimizing postoperative complications on full compliance. There is a major role of compliance in Pediatric surgical cases on a better outcome and prevention of morbidity and mortality. There are still some barriers to fully complying with the checklist despite its recommendation by WHO worldwide. We will have to improve the way of implementation with proper training and execution of the Surgical safety checklist. It is recommended to use the WHO surgical safety checklist in Pediatric Surgical cases to avoid preventable disasters in the Operating Room and improve the surgical outcome.
